# Biomedical Hydrogel Bio-Adhesive Based on Lactobionic Acid Conjugated Polyethylenimine and Oxidized Dextran with Antioxidant Activity and Cytocompatibility

**DOI:** 10.3390/pharmaceutics18080986

**Published:** 2026-08-10

**Authors:** Lei Nie, Xiaoran Hu, Shichang Cheng, Yingying Liang, Ling Wang, Wei Guo

**Affiliations:** 1 College of Life Sciences, Xinyang Normal University, Xinyang 464000, China; 2 Dabie Mountain Laboratory of Henan Province, Xinyang 464000, China

**Keywords:** hydrogels, polyethylenimine, dextran, bio-adhesives

## Abstract

**Background/Objectives:** Tissue bio-adhesives have gained significant attention as efficient alternatives to conventional wound closures, which are often hindered by insufficient adhesion and poor biocompatibility. **Methods:** Inspired by nature’s robust wet-adhesion strategies that use dynamic covalent interactions, we have reported a facilely fabricated hydrogel bio-adhesive based on lactobionic acid-conjugated polyethylenimine (LA-PEI) and oxidized dextran (ODex) via Schiff base linkages. **Results:** The prepared hydrogels exhibited three-dimensional interconnected porous networks, regulated swelling ratios, typical viscoelasticity, shear-thinning behavior, and self-healing ability. Notably, the swelling ratios of the hydrogels depended on composition, and OLP11 displayed the highest swelling ratio of over 1500%. The hydrogel bio-adhesives exhibited strong adhesion to various surfaces, including glass, metal, plastic, rubber, and wood, as well as to different chicken organs, including the heart, liver, spleen, and stomach. Furthermore, the hydrogels exhibited excellent ABTS radical-scavenging activity, effective intracellular reactive oxygen species (ROS) scavenging, and good hemocompatibility, with hemolysis ratios of all hydrogels close to 0%, below the threshold of 5%. After culturing with NIH 3T3 fibroblasts, the hydrogels demonstrated good cytocompatibility and promoted cell proliferation, with cell viabilities on day 3 reaching over 90%. **Conclusions:** This design yields multifunctional hydrogel bio-adhesives, showing strong promise for wound care and tissue repair applications.

## 1. Introduction

As the primary barrier against pathogen invasion, skin integrity is essential for maintaining internal homeostasis. However, mechanical trauma, thermal injury, and chronic ulcers induced by metabolic diseases can compromise skin architecture and impair its function [1,2,3]. Globally, wound-related medical expenditures amount to tens of billions of dollars annually [4]. Dressings serve as core materials that directly interface with wounds, critically determining the quality and efficiency of healing. Conventional dressings such as gauze and cotton pads provide basic isolation but have limited functions. They cannot actively promote tissue regeneration. Moreover, they often adhere to wound surfaces and cause secondary trauma upon removal [5,6]. In contrast, hydrogel dressings possess three-dimensional porous networks and superior hydrophilic properties that retain water, creating a persistently moist healing environment, thereby reducing infection risk and accelerating repair processes. Consequently, hydrogels have emerged as a significant advancement in the development of functional wound dressings [7,8]. Among these advanced hydrogel systems, oxidized dextran (ODex) and polyethylenimine (PEI) have been widely used in wound dressings due to their distinctive chemical structures and prominent physicochemical properties [9,10].

Dextran can be selectively oxidized by sodium periodate to obtain aldehyde-modified ODex. The reactive aldehyde groups of ODex react with amino groups of polymers via Schiff base formation. This chemical crosslinking strategy enables rapid in situ gelation of hydrogel networks under physiological conditions, endowing the resulting systems with dynamically reversible network structures, injectability, and self-healing properties. Meanwhile, ODex retains the excellent biocompatibility, controllable biodegradability, and cell adhesion and proliferation-promoting capacity of native dextran [11,12,13]. Li et al. developed a CS/ODex/halloysite nanotube (HNT) composite hydrogel based on ODex and chitosan. The hydrogel gelled in situ within 1 s, adapted to wound shapes, and showed good hemostatic and antibacterial effects [14]. Wu et al. constructed a pH/ROS dual-responsive injectable glycopeptide hydrogel via phenylboronic acid-grafted ODex and caffeic acid-grafted ε-polylysine, which possessed favorable biodegradability, stable rheological properties, and self-healing capacity and spatiotemporally controlled drug release functionality [15]. Ding et al. fabricated a dual-crosslinked photocurable hydrogel composed of ODex and methacrylated gelatin (GelMA), which featured high light transmittance, excellent compressive strength, tunable degradation behavior, and enhanced tissue adhesion [16].

PEI is a cationic polymer with densely distributed protonated amino groups along its chains. These groups disrupt bacterial cell membranes through electrostatic interactions, giving PEI-based hydrogels intrinsic antibacterial activity. In addition, the sustained release of ammonium ions from PEI neutralizes the acidic wound microenvironment and promotes fibroblast migration and proliferation [17,18,19]. Qin et al. utilized the protonated amino groups of PEI to electrostatically capture pathogens and inflammatory mediators, endowing the polysaccharide/PEI composite hydrogel with broad-spectrum antibacterial and synergistic anti-inflammatory efficacy [20]. Fang et al. prepared a CPH hydrogel by physically crosslinking carboxymethyl chitosan (CMCS) and PEI. The addition of PEI improved the inhibition rates against *Staphylococcus aureus* and *Escherichia coli* and also enhanced wet-state adhesion [21]. Furthermore, hydrogels fabricated from ODex and PEI combine rapid gelation, strong adhesion, antibacterial activity, and pro-regenerative capabilities, showing promise for wound dressing applications [22,23,24].

However, the high cationic charge density of PEI leads to cytotoxicity, nonspecific protein adsorption, and inflammatory risks. It also exhibits insufficient antioxidant capacity and mechanical toughness [25,26,27]. To overcome these limitations, one solution is to introduce flexible hydrophilic segments. These segments can shield the excess positive charges and also provide antioxidant functions and mechanical toughness [28,29]. While ODex/PEI-based hydrogels have been well documented for hemostatic and antibacterial applications via Schiff base crosslinking, PEI is known for its dose-dependent cytotoxicity due to its high cationic charge density, which limits its biomedical applications. Lactobionic acid (LA), a polyhydroxy organic acid composed of galactose and gluconic acid linked by a glycosidic bond, possesses good metal ion chelation and reactive oxygen species (ROS) scavenging capabilities. Conjugating LA to PEI neutralizes excess free amino groups and introduces carbohydrate-based targeting groups and polyhydroxy antioxidant structures [30,31]. This modification improves biosafety and modulates the wound microenvironment. Wang et al. synthesized LA-functionalized low-molecular-weight PEI gene delivery systems. The conjugates showed lower cytotoxicity, ASGPR-mediated hepatocyte targeting, and pH-dependent endosomal escape. These results confirm that LA modification balances the toxicity and transfection performance of PEI [32]. Li et al. prepared a PEI/polyacrylic acid/lactose-modified chitosan hydrogel. The hydrogel had low swelling, strong adhesion, and ROS scavenging. The flexible glycosidic moieties of lactose improved the mechanical toughness and microenvironment responsiveness of the PEI-based network [33].

Despite these advances, the integration of LA into ODex/PEI hydrogels for wound healing applications remains unexplored. To address these gaps, we developed a series of ODex/LA-PEI hydrogels. The hydrogels were systematically characterized for their microstructure, swelling behavior, mechanical properties, rheology, adhesion performance, antibacterial activity, antioxidant capacity, and blood and cytocompatibility. Notably, the prepared hydrogels exhibited self-healing capability, favorable mechanical properties, excellent ABTS radical-scavenging activity, and potent intracellular ROS-scavenging activity. They also had satisfactory hemocompatibility and cytocompatibility. These indicate their potential for wound dressing applications.

## 2. Materials and Methods

### 2.1. Chemicals

Dextran 70 (Dex), lactobionic acid (LA), polyethylenimine (PEI), and sodium periodate were purchased from Macklin Co., Ltd. (Shanghai, China). 1-ethyl-3-(3-dimethylaminopropyl)carbodiimide hydrochloride (EDC) and N-hydroxysuccinimide (NHS) were obtained from Aladdin Biochemical Technology Co., Ltd. (Shanghai, China). Triton X-100 was obtained from Amresco Co., Ltd. (Boise, ID, USA). Millipore water was prepared using a Milli-Q50 SP Reagent Water System (Millipore Corporation, Billerica, MA, USA). All reagents were obtained from commercial sources and used directly without additional purification.

### 2.2. Synthesis of Oxidized Dextran (ODex)

Dextran (4 g) was dissolved in 50 mL of deionized water with stirring until completely dissolved. Sodium periodate (4.4 g) was then added under light protection, and the reaction was stirred at room temperature in the dark for 24 h. The resulting solution was subjected to dialysis against deionized water using dialysis tubing (MWCO 3500 Da) for 5 days, with the dialysate renewed three times per day. The purified product was recovered by lyophilization and designated as ODex.

### 2.3. Synthesis of Lactobionic Acid-Modified Polyethylenimine (LA-PEI)

PEI (0.5 g) was fully dissolved in 25 mL of phosphate-buffered saline (PBS, pH 6.0) with continuous stirring. Separately, lactobionic acid (0.3 g) was dissolved in another 25 mL of PBS (pH 6.0). Then, EDC (0.08 g) and NHS (0.048 g) were added sequentially with stirring until the mixture became homogeneous. The two solutions were subsequently mixed and maintained at room temperature for 8 h to allow the reaction to proceed. The resulting solution was subjected to dialysis against deionized water using dialysis tubing (MWCO 8000–14,000 Da) for 3 days, with the dialysate renewed three times per day. The purified product was recovered by lyophilization and designated as LA-PEI.

### 2.4. Fabrication of ODex/LA-PEI Hydrogels

To prepare the OLP hydrogels, dried ODex and LA-PEI were separately dissolved in PBS (pH 7.0) at fixed volumes of 0.75 mL and 0.25 mL, respectively, in 5 mL centrifuge tubes. Each solution was vortexed until clear and then mixed to afford the hydrogel. By varying the feeding mass of ODex and LA-PEI, three hydrogel formulations were obtained: OLP11 (mass ratio ODex: LA-PEI = 1:1, 0.05 g/0.05 g), OLP13 (mass ratio ODex: LA-PEI = 1:3, 0.05 g/0.15 g), and OLP31 (mass ratio ODex: LA-PEI = 3:1, 0.15 g/0.05 g).

### 2.5. ^1^H Nuclear Magnetic Resonance (^1^H NMR) Spectroscopy

A total of 20 mg each of ODex, Dex, LA, and LA-PEI were individually dissolved in 0.5 mL of deuterated water (D_2_O). Each solution was pipetted into NMR tubes to a column height of 4 cm, and ^1^H NMR measurements were performed at room temperature using a nuclear magnetic resonance spectrometer (JNM-ECZ600R/S3, JEOL, Tokyo, Japan).

### 2.6. Fourier Transform Infrared Spectroscopy (FT-IR) Analysis

To confirm the successful preparation of the materials and hydrogels, Fourier transform infrared (FT-IR) spectroscopy was performed. Specifically, measurements were carried out on an FT-IR spectrometer (ThermoFisher Scientific, NicoletiS5, Waltham, MA, USA) fitted with an attenuated total reflectance (ATR) accessory, and the raw materials and hydrogels (Dex, ODex, LA, LA-PEI, OLP11, OLP13, and OLP31) were examined. The spectra were collected over a wavenumber range of 500–4000 cm^−1^ at a resolution of 1 cm^−1^, with each spectrum accumulated from 64 scans [34].

### 2.7. Scanning Electron Microscopy (SEM) Characterization

The wet composite hydrogel samples were frozen at −20 °C for 24 h and subsequently lyophilized for 3 d to obtain dry hydrogel samples. The dry samples were immersed in liquid nitrogen for approximately 1 min, removed, and then cut with a blade into blocks of uniform size and thickness. The resulting pieces were attached to the sample holder with conductive adhesive and sputter-coated with platinum. Morphological observation of the composite hydrogels was conducted on a Regulus 8220 scanning electron microscope (SEM, Hitachi, Tokyo, Japan) at an accelerating voltage of 30 kV [35], and the pore dimensions were determined using ImageJ software (Version 1.53).

### 2.8. Antioxidant Activity

The antioxidant activity of OLP hydrogels was evaluated by the 2,2′-azino-bis (3-ethylbenzothiazoline-6-sulfonic acid) diammonium salt (ABTS) radical scavenging assay based on a prior report [36]. To generate the radicals, 3 mL of ABTS (12 mg) was oxidized by 3 mL of potassium persulfate (2 mg) in the dark at 4 °C for 12 h, and the resultant solution was diluted 23-fold with deionized water to obtain an ABTS^+^ working solution with a final concentration of 0.17 mg/mL (approximately 0.32 mmol/L). The scavenging activity was measured by adding 0.02 g of the dried sample to 5 mL of the diluted ABTS solution, followed by shaking at 30 °C and 150 r/min for 30 min in the dark. After the mixture was allowed to stand, the supernatant was collected, and its absorbance at 734 nm was measured using a microplate reader (AMR-100, Hangzhou, China). The ABTS radical scavenging activity was determined via the equation:

$$\mathrm{ABTS}\;\mathrm{Scavenging}\;\mathrm{Activity}\;\left(\%\right)=\frac{A_{c}-A_{b}}{A_{c}}\;\times \;100\%$$

where *A_c_* denotes the absorbance of the control group, and *A_b_* denotes that of the sample group.

### 2.9. Swelling Behavior Test

To evaluate the water absorption of the composite hydrogels, a gravimetric method was employed at ambient temperature. Dried hydrogel samples (10 mg each) were placed in 5 mL of PBS (pH 7.4) and kept at room temperature for 36 h until equilibrium swelling was attained. The time point at 36 h was selected based on preliminary swelling kinetic studies, in which the weight change of all hydrogel formulations had already become minimal after 24 h, with only negligible changes. After excess surface water was removed with filter paper, the weight of the swollen samples (*m*_1_) was measured. The equilibrium swollen sample was subsequently freeze-dried for 3 d, and the dry mass (*m*_2_) was recorded. The swelling ratio was determined via the equation:

$$\mathrm{SR}(\%)\;=\;\frac{m_{1}-m_{2}}{m_{2}}\times 100\%$$

### 2.10. Rheological Characterization Test

Rheological characterization of the hydrogel specimens was performed at 37 °C employing a TA rheometer (DHR-2, New Castle, DE, USA). A constant strain of 1% and a fixed angular frequency of 10 rad/s were applied, during which the storage modulus (G′) and loss modulus (G″) were acquired via an aluminum parallel-plate geometry (20 mm in diameter). To minimize moisture loss during the measurement, silicone oil was utilized to coat the rim of the geometry. Afterward, oscillatory strain sweep measurements were conducted from 0.1% to 500% strain at an angular frequency of 10 rad/s to identify the linear viscoelastic region. Finally, the frequency sweep experiments were implemented under a constant strain of γ = 0.5%.

### 2.11. Adhesion Performance Test

To characterize the adhesion behavior of the OLP hydrogels, wet hydrogel samples were mounted on various substrates, including glass, metal, plastic, rubber, and wood. Given their potential usage in living systems [37], adhesion assessments were further carried out on different chicken organs (namely, the heart, liver, spleen, and stomach). The procedures involving laboratory animals were approved by the Ethics Committee of Xinyang Normal University (approval code: XFEC-2026-074).

### 2.12. Antibacterial Activity Test

To investigate the antimicrobial capacity, *Staphylococcus aureus* (*S. aureus*) and *Escherichia coli* (*E. coli*) were chosen as representative bacterial models [38]. The cryopreserved strains (−80 °C) were activated by streaking, and a single colony was subsequently isolated and transferred into LB broth for overnight incubation at 37 °C under orbital agitation (180 rpm) to promote logarithmic growth. The resulting bacterial culture was then serially diluted with sterile 0.9% saline to attain an optical density at 600 nm (OD_600_) ranging from 0.6 to 0.8. A pre-measured 0.015 g hydrogel sample was placed into a sterile glass vessel containing 10 mL of LB broth, followed by the addition of 10 µL of the adjusted bacterial suspension, then vortexed. This mixture was subsequently maintained in a shaker at 37 °C and 150 rpm for 6 h. The post-incubation OD_600_ values were recorded utilizing a microplate reader. The antibacterial ratio was determined via the equation:

$$\mathrm{Antibacterial}\;\mathrm{Ratio}\;(\%)\;=\;\frac{k_{b}-k_{s}}{k_{b}}\times 100\%$$

where *k_b_* and *k_s_* denote the absorbance of the control and hydrogel-treated groups, respectively.

Ultimately, 20 µL of the culture broth from both groups was uniformly spread onto nutrient agar plates. These plates were inverted and incubated statically at 37 °C in a biochemical chamber for 12 h. Following this period, bacterial colonies were visually inspected and documented to further elucidate the bactericidal performance of the hydrogel.

### 2.13. Biocompatibility Evaluation

To examine the in vitro biocompatibility of the OLP hydrogel, the Cell Counting Kit-8 (CCK-8) method was utilized to evaluate cytotoxic effects on murine NIH-3T3 fibroblasts [39]. For extract preparation, the hydrogel specimens were initially subjected to a 12 h sterilization process via submersion in 75% ethanol combined with UV light, followed by multiple sterile PBS rinses to ensure the complete removal of alcohol residues. Subsequently, each pre-sterilized sample was immersed in an equivalent volume of complete DMEM (enriched with 10% fetal bovine serum and 1% penicillin-streptomycin), and the resulting mixture was incubated for 24 h at 37 °C to collect the conditioned medium. NIH-3T3 cells in the exponential phase were collected and transferred into 96-well plates at a density of 5 × 10^3^ cells/well (100 µL per well). The cells were then co-incubated with the acquired hydrogel extract under a humidified, 5% CO_2_ atmosphere at 37 °C over a period of 1, 2, and 3 days. Blank wells containing only culture medium and experimental wells containing the OLP hydrogel were established as controls. At each designated interval, the CCK-8 reagent was supplemented at a final concentration of 10% (*v*/*v*) relative to the culture volume, followed by an additional 2 h incubation at 37 °C. Finally, the optical density (OD) at 450 nm was recorded using a Spectra Max 190 microplate reader (Molecular Devices, Berlin, Germany) [40]. The cell viability rates were then determined using the standard equation:

$$Cell\;Viability\left(\%\right)=\frac{A_{s}\;-\;A_{b}}{A_{c}\;-\;A_{b}}\;\times \;100\%$$

where *A_s_*, *A_c_*, and *A_b_* denote the optical density values acquired from the experimental sample group, the negative control group, and the blank group, respectively.

### 2.14. Intracellular ROS Scavenging Assay

To quantify the intracellular ROS scavenging efficacy of the hydrogel, a Reactive Oxygen Species Assay Kit (DCFH-DA, Beyotime, Shanghai, China) was employed. Briefly, NIH-3T3 cells were inoculated into culture plates at a density of 3 × 10^5^ cells per well and maintained for 24 h. A peroxide solution was then introduced to stimulate the cells. After removing the initial culture medium and rinsing the adherent cells twice with PBS, the specimens were assigned to three distinct groups: a blank control (cells cultured in medium), a negative control (cells treated with the H_2_O_2_ dilution), and a treatment group (cells co-treated with H_2_O_2_ and the hydrogel extract). All groups were subsequently kept in a light-protected environment for 2 h. Upon completion of this incubation, the treatment liquids were discarded, and the cells underwent three thorough PBS rinses to thoroughly eliminate any remaining H_2_O_2_. Thereafter, a pre-diluted DCFH-DA staining solution was introduced into the wells, followed by an additional 30 min of dark incubation. After staining, the excess fluorescent probes were washed off by rinsing the cells three times with PBS. Ultimately, the stained cells were visualized and photographed under a fluorescence microscope, and the intracellular ROS levels were further quantified based on the recorded green fluorescence intensities.

### 2.15. Fluorescent Live/Dead Staining

Murine NIH-3T3 fibroblasts (CRL-1658^TM^, ATCC) were inoculated onto 35 mm Petri dishes and incubated in the presence of the prepared hydrogel extracts. Cells cultured in complete medium without any hydrogel extract served as the control group. On days 1, 2, and 3, the culture medium was discarded, and the adherent cells were stained using a live/dead cell viability assay kit. Calcein-AM was excited at 488 nm with emission at 515 nm (FITC channel), while PI was excited at 548 nm with emission at 564 nm (Cy3 channel). Fluorescent micrographs were subsequently captured via a confocal laser scanning microscope, where viable cells exhibited green fluorescence, whereas dead cells appeared red.

### 2.16. Cell Scratch Assay

A sterile Ibidi Culture-Insert (2-well) was aseptically placed into 6-well plates. Next, 5 × 10^4^ NIH-3T3 cells suspended in 80 µL were inoculated into each insert well. Following 24 h of incubation to reach confluence, a cell-free gap (~500 µm) was generated by carefully removing the inserts, and the monolayers were gently rinsed with PBS. Cells were then maintained with hydrogel extract (containing 1% FBS) or complete medium (control) for 6 and 12 h. Wound closure was photographed at 0, 6, and 12 h [41]. ImageJ (v1.53) was utilized to quantify the migration rate via the following formula:

$$\mathrm{Migration}\;\mathrm{Rate}\;\left(\%\right)\;=\;\frac{S_{0}-S_{n}}{S_{n}}\;\times \;100\%$$

where *S*_0_ represents the initial scratch width, and *S_n_* represents the width of the scratch at 6 h and 12 h.

### 2.17. Hemolysis Evaluation

Sterile sheep blood anticoagulated with EDTA was subjected to centrifugation at 3000 rpm for 10 min to separate the erythrocytes. The RBCs were then rinsed with normal saline (blood: saline ratio of 1:1.25), re-centrifuged under identical conditions, and resuspended to obtain a working RBC suspension. For the test, 2.5 mg of lyophilized hydrogel was incubated with 1 mL of saline at 37 °C for 30 min, followed by the addition of 20 µL of RBC suspension as the experimental group (*A_s_*). Deionized water (1 mL) and normal saline (1 mL) were mixed with 20 µL RBCs to serve as the positive (*A_p_*) and negative (*A_n_*) controls, respectively. All groups were further maintained at 37 °C for 1 h and centrifuged. Finally, the supernatants were harvested, and the optical density at 540 nm was recorded via a microplate reader [42]. The hemolysis rate is calculated using the following equation:

$$Hemolysis\;rate(\%)=\frac{A_{s}-A_{n}}{A_{p}-A_{n}}\;\times \;100\%$$

### 2.18. Statistical Analysis

All quantitative assays were carried out in at least three independent replicates. The results are presented as mean ± standard deviation (SD). GraphPad Prism 10.0 was used to perform one-way ANOVA for the hemolysis and ROS data, and two-way ANOVA for the CCK-8 results. All remaining experimental data were subjected to one-way ANOVA via SPSS 28.0, followed by Tukey’s post hoc test. Statistical significance was defined as * *p < 0.05*, ** *p < 0.01*, *** *p < 0.001*, and **** *p < 0.0001*; ‘ns’ indicates no significant difference.

## 3. Result and Discussion

### 3.1. Fabrication of OLP Hydrogels

**Figure 1:**
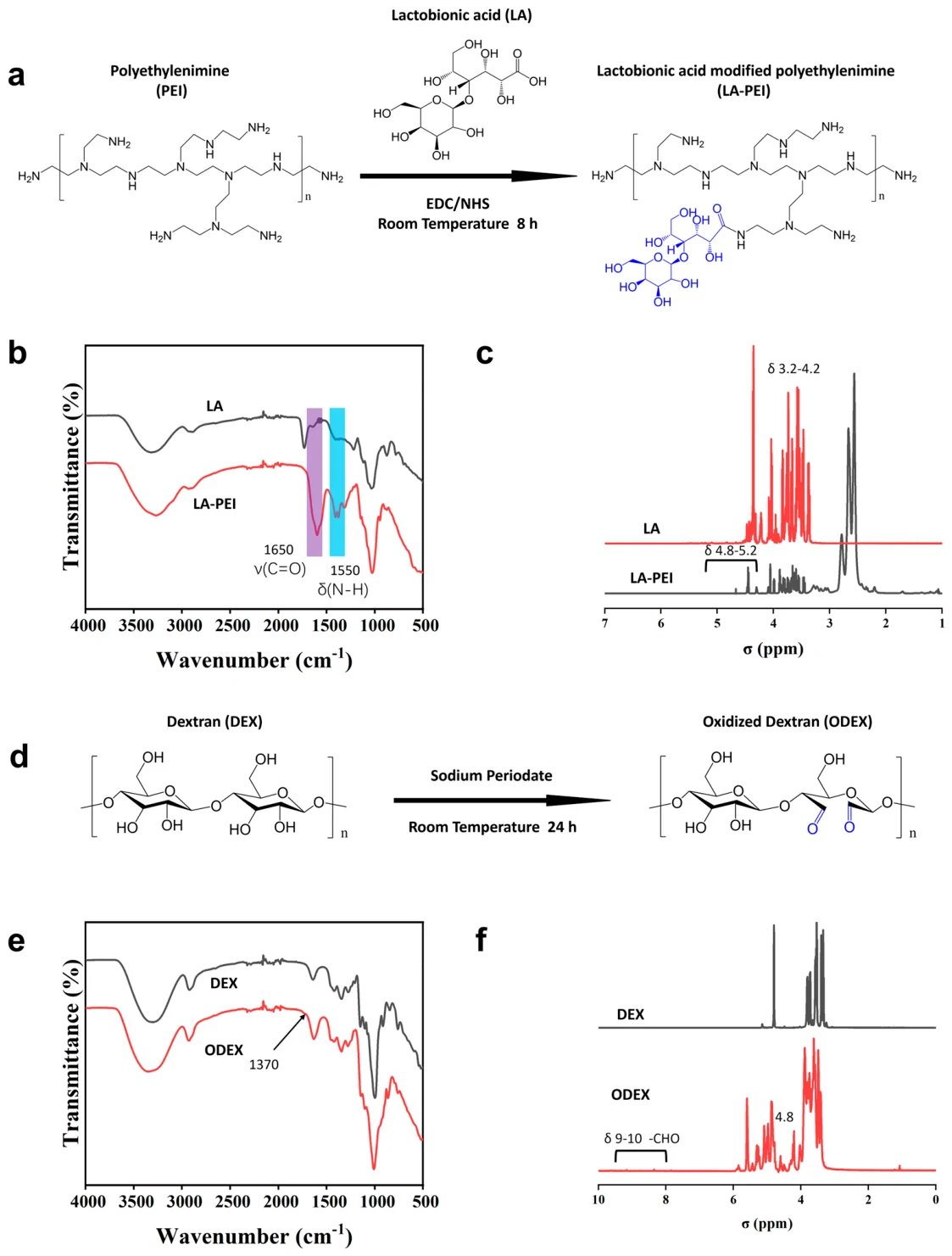
Synthesis pathways of LA-PEI (**a**) and ODex (**d**). FT-IR spectra of LA-PEI (**b**) and ODex (**e**). ^1^H NMR spectra of LA-PEI (**c**) and ODex (**f**).

LA-PEI was synthesized via EDC/NHS-catalyzed amidation between the carboxyl groups of lactobionic acid (LA) and the amine groups of polyethyleneimine (PEI), while ODex was prepared through the selective oxidation of dextran using sodium periodate (Figure 1a,d). The amidation reaction created stable amide linkages, and the periodate oxidation introduced reactive aldehyde groups, thereby providing the essential building blocks for the subsequent fabrication of Schiff-base crosslinked hydrogels. To verify the success of the reactions and analyze the structural transformations, we conducted FT-IR (Figure 1b,e) and ^1^H NMR characterizations (Figure 1c,f) on the raw materials (LA, PEI, and dextran) as well as the synthesized LA-PEI and ODex. In the FT-IR spectrum of LA-PEI (Figure 1b), the characteristic absorption peaks at 1650 cm^−1^ and 1550 cm^−1^ were attributed to the stretching vibration of C=O (amide I) and the bending vibration of N-H (amide II), respectively. Moreover, the ^1^H NMR spectrum of LA-PEI (Figure 1c) displayed the typical resonance peaks of LA in the δ 3.2–4.2 ppm range, corroborating the successful grafting of LA onto the PEI chains. For ODex, the FT-IR spectrum (Figure 1e) exhibited a distinctive variation at 1370 cm^−1^, which was characteristic of the aldehyde C-H deformation. Crucially, the ^1^H NMR spectrum of ODex (Figure 1f) showed a newly emerged characteristic signal at δ 9–10 ppm, which indicates the aldehyde proton (-CHO), providing definitive evidence for the successful oxidation of dextran. Overall, the combined appearance of characteristic amide and aldehyde signals in the LA-PEI and ODex precursors provided primary evidence of successful chemical modification of the functional building blocks, confirming their potential for subsequent preparation of dynamic covalent hydrogel networks.

**Figure 2:**
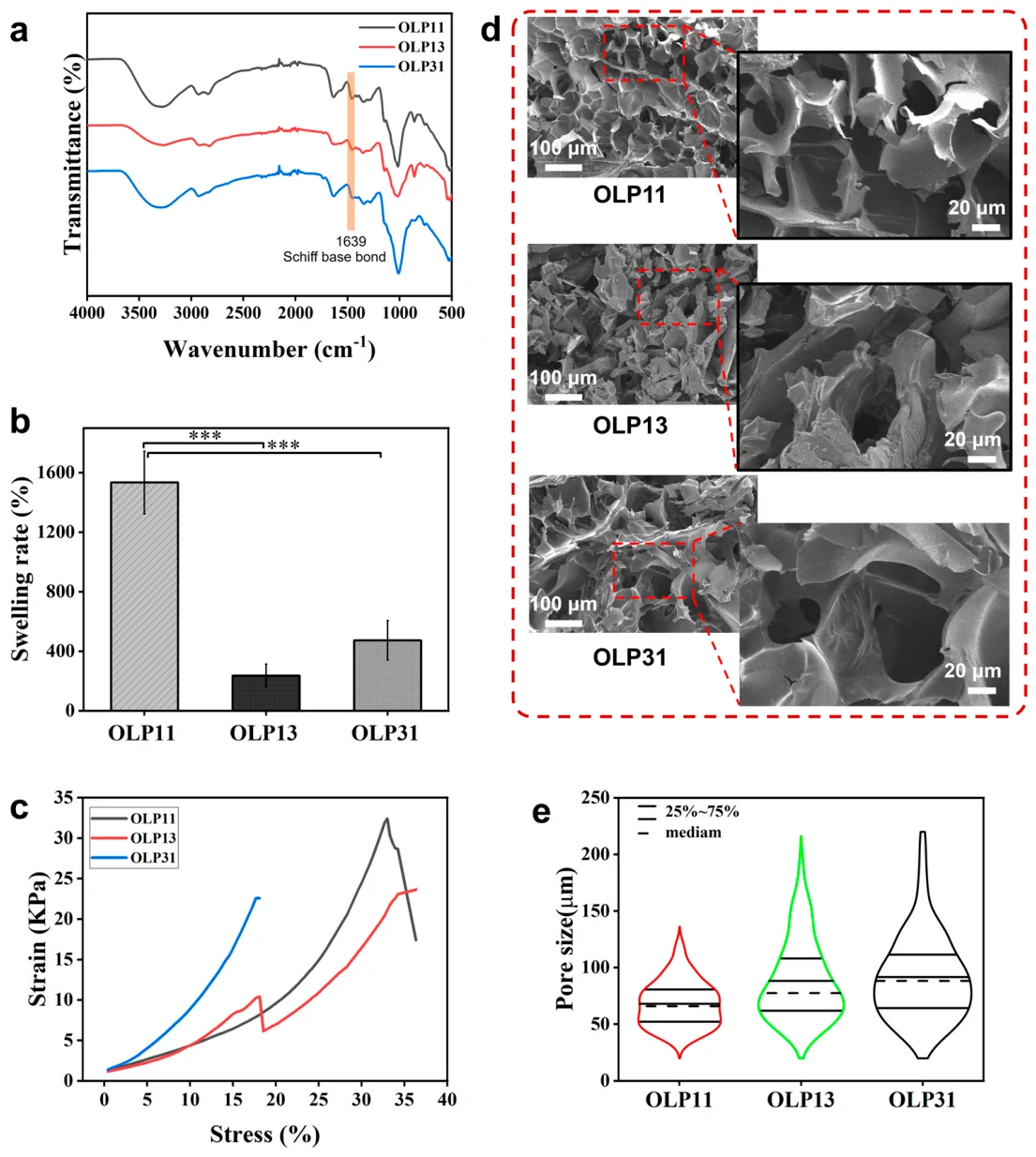
Physicochemical and microstructural characterization of OLP hydrogels. (**a**) FT-IR spectra showing the characteristic Schiff base peak at 1639 cm^−1^. (**b**) Equilibrium swelling ratios in PBS. (**c**) Representative tensile stress–strain curves. (**d**) SEM images of the lyophilized hydrogels reveal interconnected porous networks. Scale bars: 100 μm and 20 μm. (**e**) Violin plots of pore size distribution. Statistical significance is denoted by asterisks (*** *p* < 0.001).

FT-IR spectroscopy was employed to characterize the chemical crosslinking behavior of OLP hydrogels fabricated with varied mass ratios of ODex to LA-PEI, by comparing the transmittance spectra of OLP11, OLP13 and OLP31 (Figure 2a). It was observed that all three OLP samples displayed a characteristic absorption band at 1639 cm^−1^, highlighted by the orange shaded area, which corresponded to the C=N stretching vibration of Schiff base bonds, thus confirming the successful covalent crosslinking between aldehyde groups of ODex and amine groups of LA-PEI [43]. The Schiff base crosslinking proceeds through the nucleophilic addition–elimination reaction between aldehyde moieties on ODex and primary amine groups on LA-PEI. Simultaneously, a broad transmittance depression spanning 3200–3600 cm^−1^ was detected for all samples, originating from the stretching vibrations of residual -OH and -NH- groups. Notably, the absorption intensity of the 1639 cm^−1^ Schiff base peak follows the sequence OLP31 > OLP13 > OLP11, revealing that the ODex/LA-PEI mass ratio of 3:1 promotes the generation of more C=N linkages [44]. In the fingerprint region of 1000–1500 cm^−1^, OLP31 exhibited much deeper absorption valleys relative to OLP11 and OLP13, which was ascribed to the higher contents of C–O and C–N single bonds introduced by excess ODex, consistent with previous FT-IR characterization of polysaccharide–polyamine crosslinked hydrogels [45].

The equilibrium swelling behavior of OLP11, OLP13, and OLP31 hydrogels was assessed in PBS buffer, and the corresponding swelling rates are displayed in Figure 2b. The swelling performance of the OLP11 hydrogel was significantly higher than that of OLP13 and OLP31. Such distinctions were mainly attributed to the varied crosslinking densities regulated by different ODex/LA-PEI mass ratios.

Next, tensile tests were performed on fully swollen hydrogels, and the corresponding curves are presented in Figure 2c. OLP31 displayed a nearly linear ascending curve without obvious fluctuations, while OLP11 and OLP13 showed irregular nonlinear profiles during stretching. OLP31 reached its maximum strain at low stress, and OLP11 bore the ultimate stress before fracture, with OLP13 showing intermediate mechanical behavior. Such distinct mechanical behaviors were determined by the tunable Schiff-base crosslink network formed at different ODex/LA-PEI ratios. Higher crosslink density in OLP31 restricted chain sliding and imparted linear-elastic characteristics, whereas the looser crosslink network in OLP11 enhanced stretchability. This correlation between crosslink density and tensile behavior was consistent with previous studies on swollen polysaccharide–imine crosslinked hydrogels [46,47].

### 3.2. Microstructure and Water Absorption Properties of OLP Hydrogels

The physicochemical properties of OLP hydrogels were investigated, including microstructure, swelling capability, rheological characteristics, self-healing ability, and adhesion performance. The morphological characteristics of freeze-dried OLP11, OLP13, and OLP31 were observed by SEM (Figure 2d). All OLP hydrogels present a porous and interconnected three-dimensional network structure, facilitating fluid absorption, nutrient exchange, and cell migration, which suggests their appropriateness for diverse biomedical applications, such as wound dressing, bio-adhesives, and tissue engineering [48]. OLP11 exhibited a dense network with small, uniform pores. OLP13 displayed an intermediate pore size. Quantitative analysis of pore sizes (OLP11, *n* = 56; OLP13, *n* = 58; OLP31, *n* = 67) revealed mean pore sizes of 68.01 μm, 88.12 μm, and 91.56 μm, respectively. In contrast, OLP31 possessed significantly enlarged macropores with a broader distribution range, as evidenced by a higher median pore size (88.36 μm) compared to OLP11 (65.04 μm), and a substantially wider full range (35.79–214.87 μm, span = 179.08 μm) relative to that of OLP11 (39.16–117.45 μm, span = 78.29 μm), representing a 2.3-fold expansion in pore size span, which was consistent with statistical results from violin plots (Figure 2e).

These structural variations were primarily governed by the crosslinking density. In OLP11, the high crosslinking density severely restricted water molecule mobility during freezing. Consequently, ice crystal coarsening was effectively inhibited, resulting in fine and homogeneous pores [43,45]. Conversely, the excess aldehyde groups in OLP31 enhanced the network’s hydrophilicity. They also introduced topological heterogeneity within the Schiff-base crosslinks. This weakened the physical constraint on ice expansion. Thus, large ice crystals formed and left behind enlarged macropores after lyophilization [38]. This tunable porous architecture offers distinct advantages for biomedical engineering. Large macropores facilitate efficient nutrient diffusion and cell migration. Meanwhile, smaller pores provide enhanced mechanical stability. Therefore, the OLP hydrogels can be tailored to meet the specific requirements of various tissue repair applications [49].

### 3.3. Rheological Behavior and Self-Healing Ability of OLP Hydrogels

**Figure 3:**
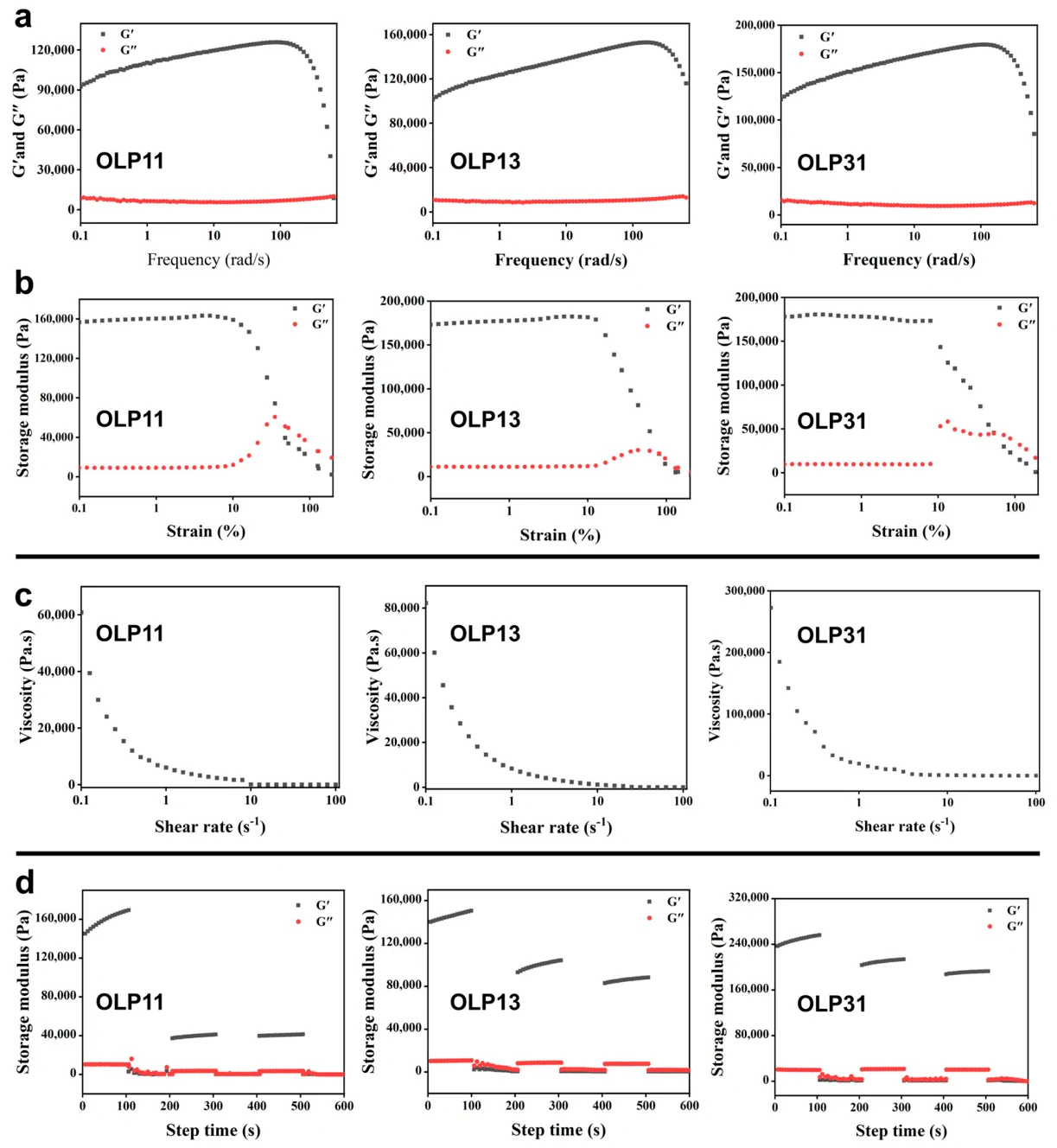
The storage modulus (G′) and loss modulus (G″) of OLP hydrogels in terms of (**a**) frequency and (**b**) strain. (**c**) The rheological behavior of OLP hydrogels. (**d**) The self-healing capability of OLP hydrogels was evaluated using 3-cycle step strain tests.

Evaluation of the rheological properties of OLP hydrogels, particularly the storage modulus (G′) and loss modulus (G″), is critical for predicting their tissue-wetting behavior. Through rheometer testing, the moduli of the OLP specimens were systematically recorded under varying frequency and strain conditions (Figure 3a,b). Strain sweep measurements (Figure 3b) revealed that the storage modulus remained stable below 10% strain, confirming the linear viscoelastic region (LVR). Accordingly, 1% strain, located well within the LVR, was adopted for subsequent frequency sweep and cyclic strain tests to prevent artificial destruction of the hydrogel network. Across all these parameters, the G′ values consistently exceeded those of G″, thus demonstrating a prominent viscoelastic response typical of hydrogel networks. With escalating frequency and strain, a pronounced drop in G′ was observed, eventually crossing over the ascending G″, which signified the structural disruption of the hydrogel matrices. Additionally, as shown in Figure 3c, all hydrogel groups exhibited a steep decrease in apparent viscosity over shear rates ranging from 0.1 to 100 s^−1^, indicating non-Newtonian pseudoplastic behavior. Such shear-thinning traits are pivotal for enabling minimally invasive administration and immediate in situ gelation. Considering the weak mechanical properties of OLP hydrogels, tensile measurements could not be performed in this study.

Furthermore, the self-healing behavior of OLP hydrogels was investigated by step-strain rheological tests (Figure 3d). The self-healing properties are attributed to the dynamic nature of the imine bonds (C=N) [38]. Under mechanical stress, these reversible bonds break to dissipate energy. Upon stress removal, the bonds rapidly reform, restoring the network integrity. These characteristics make OLP hydrogels highly promising as injectable biomaterials for minimally invasive wound healing applications.

### 3.4. Adhesion Performance of Hydrogels

**Figure 4:**
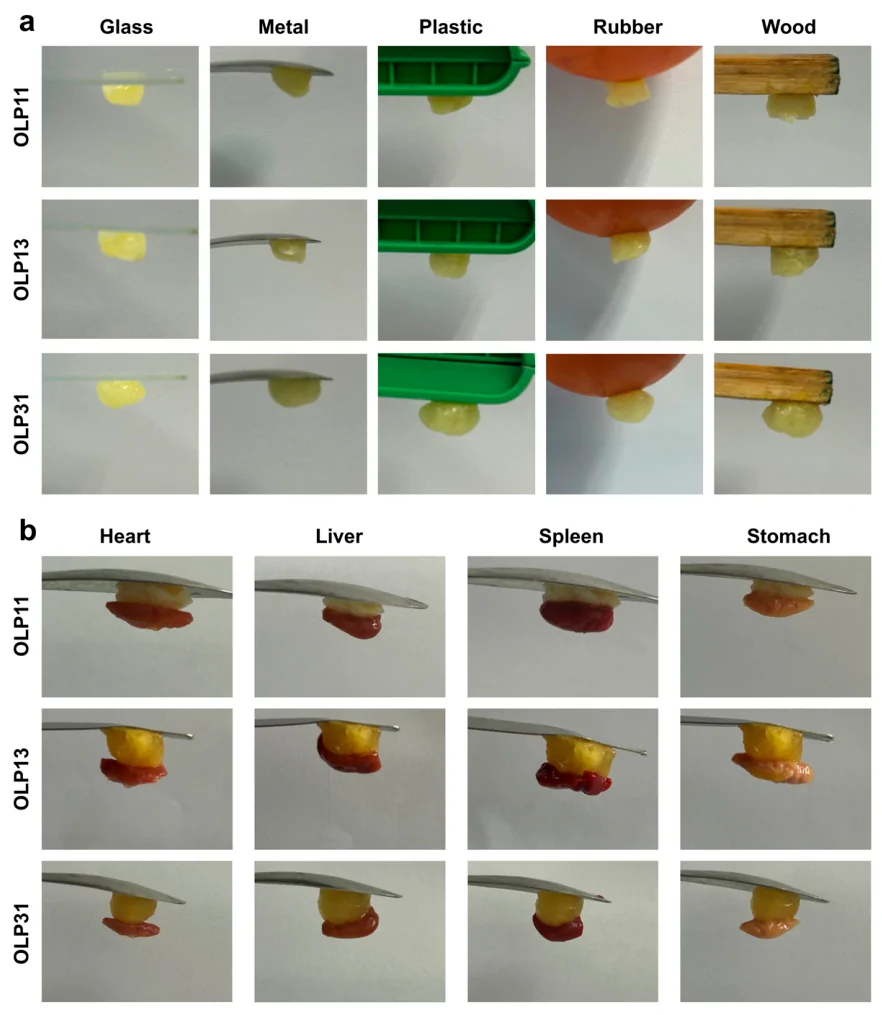
Photographs showing the adhesion of OLP hydrogels to glass, metal, plastic, rubber, and wood are presented in (**a**), while (**b**) displays images of hydrogel adhesion to various organs, including heart, liver, spleen, and stomach.

The adhesion performance of the hydrogels was systematically investigated using various substrates, including synthetic materials and biological tissues. As presented in Figure 4a, the hydrogels with different component ratios adhered strongly and durably to glass, metal, plastic, rubber, and wood. In parallel, Figure 4b illustrated that these hydrogels, as potential biomedical materials, also exhibited superior tissue adhesion, showing firm bonding to chicken heart, liver, spleen, and stomach [50]. The superior adhesive properties of these hydrogels are primarily derived from the synergistic effect of chemical bonding and physical interactions. At the chemical level, the aldehyde groups (-CHO) along the oxidized dextran chains can reversibly react with the residual primary amine groups (-NH_2_) of ethylene imine polymer (PEI) via Schiff base formation, generating covalent cross-links that confer robust adhesion properties to the hydrogels [51]. At the physical level, PEI, being a cationic polymer, exhibits strong electrostatic interactions between its protonated amine groups and the negatively charged cell membrane surfaces, facilitating rapid initial adhesion. The synergistic effect of the aforementioned physical and chemical interactions confers robust adhesion properties on the hydrogels.

### 3.5. Antioxidant and Antibacterial Properties of Hydrogels

**Figure 5:**
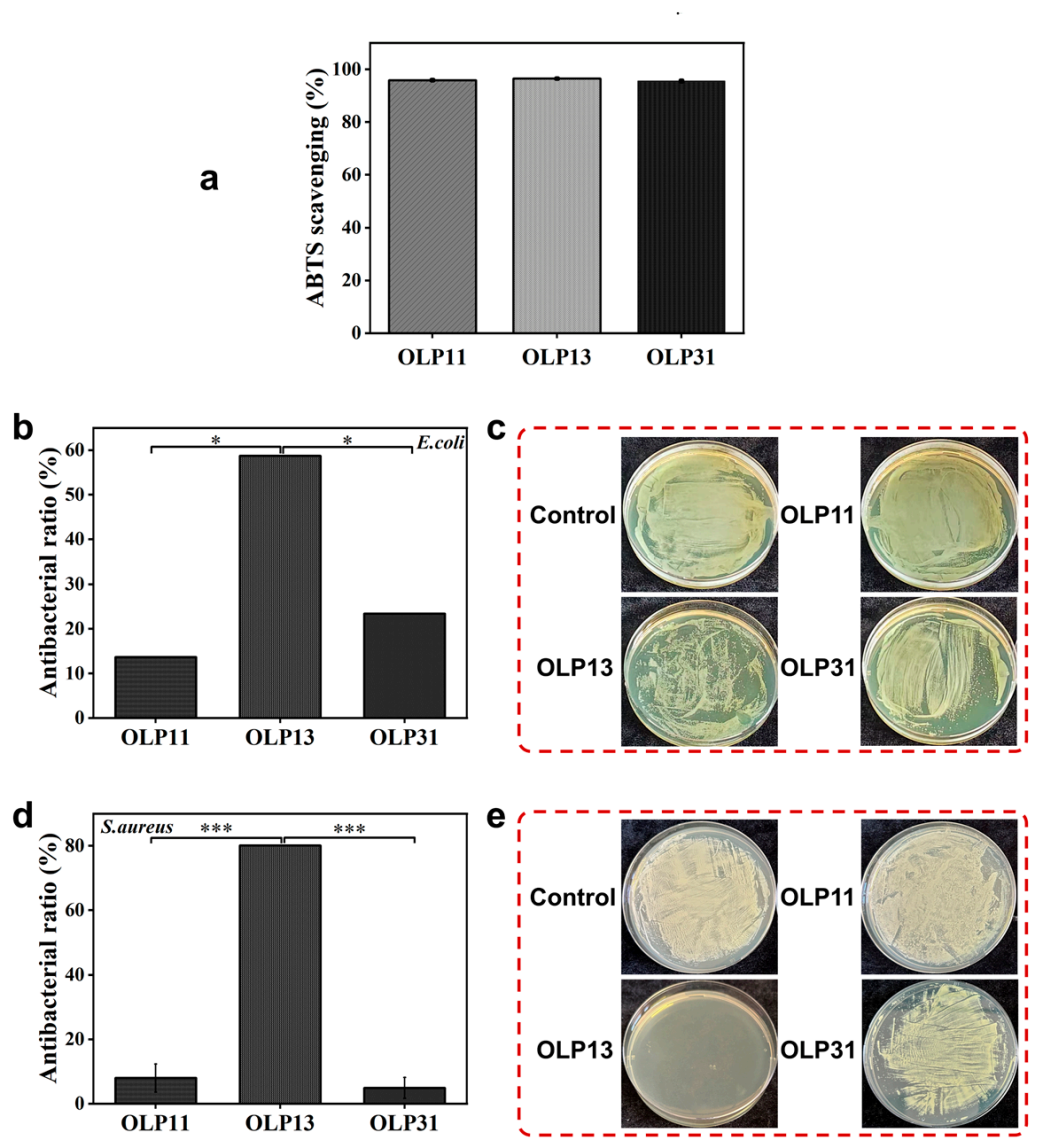
(**a**) Free radical scavenging capacity of hydrogels, as determined by the ABTS assay. (**b**) The antibacterial ratios of hydrogels against *E. coli.* (**d**) The antibacterial ratios of hydrogels against *S. aureus.* (**c**) Photographs showing hydrogels co-cultured with E. coli on nutrient agar (NA) plates. (**e**) Photographs showing hydrogels co-cultured with S. aureus on nutrient agar (NA) plates. The antibacterial ratios of hydrogels against (**b**) *E. coli* and (**d**) *S. aureus* are presented. Statistical significance: * *p* < 0.05, *** *p* < 0.001.

Post-traumatic inflammatory responses and ischemia–reperfusion injury often lead to a massive burst of reactive oxygen species (ROS), which can cause oxidative damage to cell membranes and intracellular biomacromolecules, resulting in secondary tissue damage. Therefore, endowing hydrogels with favorable antioxidant properties to scavenge excess ROS is a key strategy for alleviating oxidative stress and accelerating wound healing. The antioxidant capacity of the OLP hydrogels was evaluated by measuring the ABTS radical scavenging activity. As shown in Figure 5a, the ABTS scavenging rates in all hydrogel-treated groups exceeded 90%, compared with the control group. This can be primarily attributed to the synergistic effects of the electron-donating properties of amine groups, the hydrogen atom-donating ability of aldehyde groups, and the stabilizing action of multiple hydroxyl groups, which collectively promoted the conversion of free radicals into neutral molecules. After disruption of the skin barrier, the wound is highly susceptible to invasion by external pathogenic microorganisms. Bacterial proliferation can trigger local infection, prolong the inflammatory phase, and significantly delay the healing process. Endowing hydrogels with antibacterial properties can enhance their repair efficacy. The antibacterial activity of the synthesized hydrogels was evaluated against Gram-negative bacteria (*E. coli*) and Gram-positive bacteria (*S. aureus*). As shown in Figure 5b,d, the hydrogels exhibited certain inhibitory effects on the growth of both *E. coli* and *S. aureus*. Specifically, the inhibition rates of OLP11, OLP13, and OLP31 against *E. coli* were 13.66%, 58.70%, and 23.39%, respectively, while those against *S. aureus* were 7.99%, 80.08%, and 4.89%, respectively. After co-incubating the hydrogel samples with the bacterial suspension for 6 h, 20 μL of the culture was evenly spread onto nutrient agar (NA) plates and incubated for an additional 12 h. As shown in Figure 5c,e, the number of bacterial colonies in the hydrogel-treated groups was significantly reduced compared with the blank control group. The antibacterial activity of the hydrogels against both bacterial strains is attributed to the synergistic effect of electrostatic adsorption mediated by the positive charges of PEI and Schiff base crosslinking involving aldehyde groups [52]. These two mechanisms collectively compromise the structural integrity of the bacterial surface, leading to cytoplasmic leakage and consequently resulting in bacterial inhibition.

### 3.6. Hemolysis and In Vitro Cellular ROS Scavenging Performance of Hydrogels

**Figure 6:**
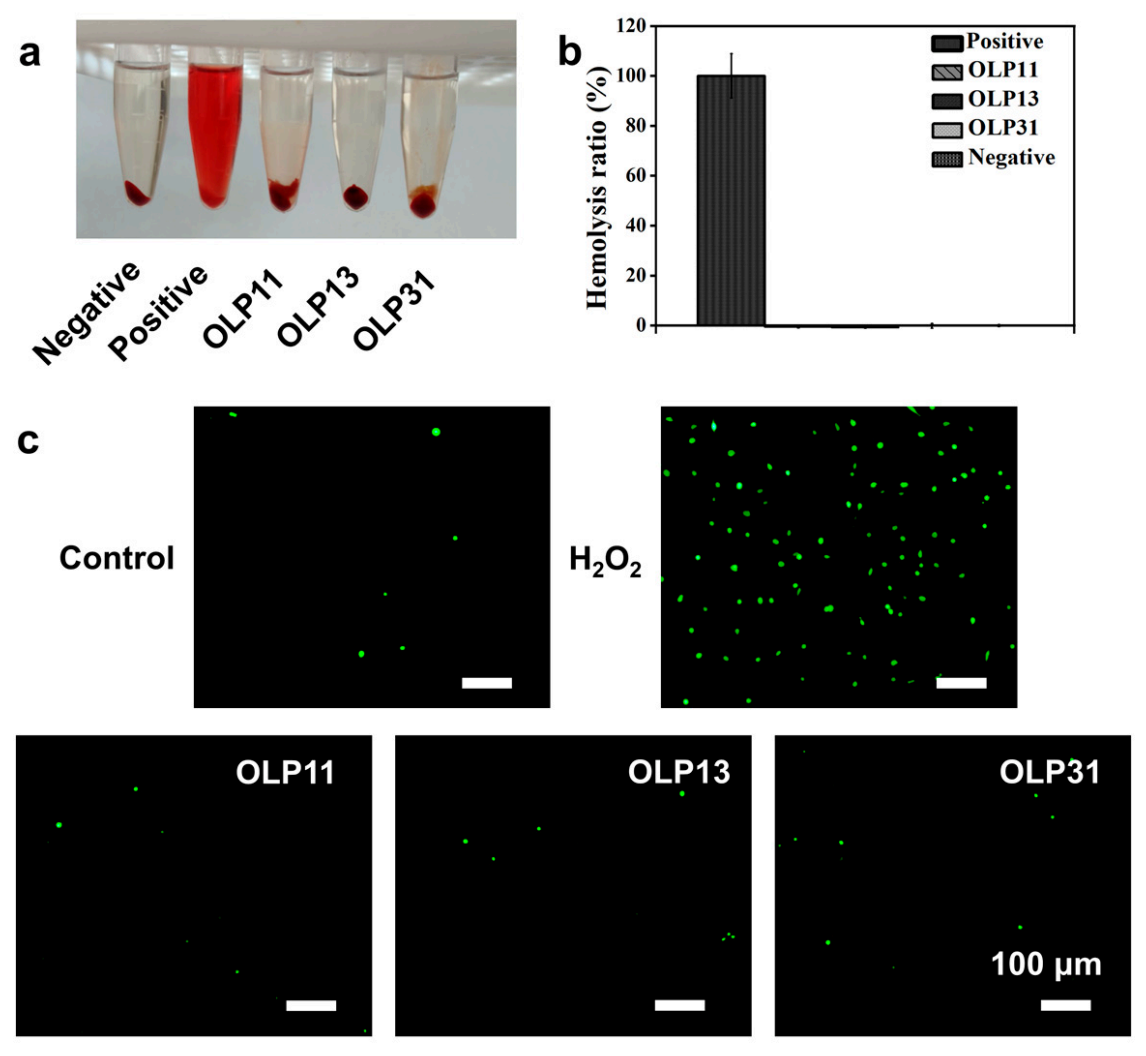
(**a**,**b**) Hemolysis assay of OLP hydrogels. (**c**) DCFH-DA staining assay to characterize the intracellular ROS scavenging ability of hydrogels.

We evaluated the hemocompatibility of the hydrogels. Good hemocompatibility is a prerequisite for wound dressing applications. According to the standards established by the U.S. Food and Drug Administration (FDA), the acceptable hemolysis ratio for biomaterials is less than 5% [53]. As illustrated in Figure 6b, the hemolysis ratios of all hydrogel groups were below 5%, which was well within the acceptable limit stipulated by the FDA. Compared with the negative control group treated with 0.9% normal saline, the supernatant color of the hydrogel-treated groups exhibited no significant difference (Figure 6a), demonstrating the favorable blood compatibility of the hydrogels. The low hemolysis ratio of the hydrogels may be attributed to the effective shielding of excessive positive charges on the ethylene imine polymer chains by lactobionic acid grafting, thereby reducing the electrostatic interactions that damage red blood cell membranes. As presented in Figure 6c, the DCFH-DA probe was employed to evaluate intracellular ROS levels and thereby assess the regulatory effects of hydrogels with varying ratios on oxidative stress. The positive control group treated with H_2_O_2_ showed intense green fluorescence, indicative of substantially elevated ROS levels, whereas the fluorescence intensity in all hydrogel treatment groups tended to decrease. The decrease in DCFH-DA fluorescence intensity may be attributed to the synergistic ROS-scavenging effects of the residual aldehyde groups, dynamic Schiff base bonds, and the amine-rich PEI backbone within the hydrogels. These active moieties collectively consumed intracellular reactive oxygen species that would otherwise oxidize non-fluorescent DCFH to green-fluorescent DCF.

### 3.7. Cytocompatibility of OLP Hydrogels

**Figure 7:**
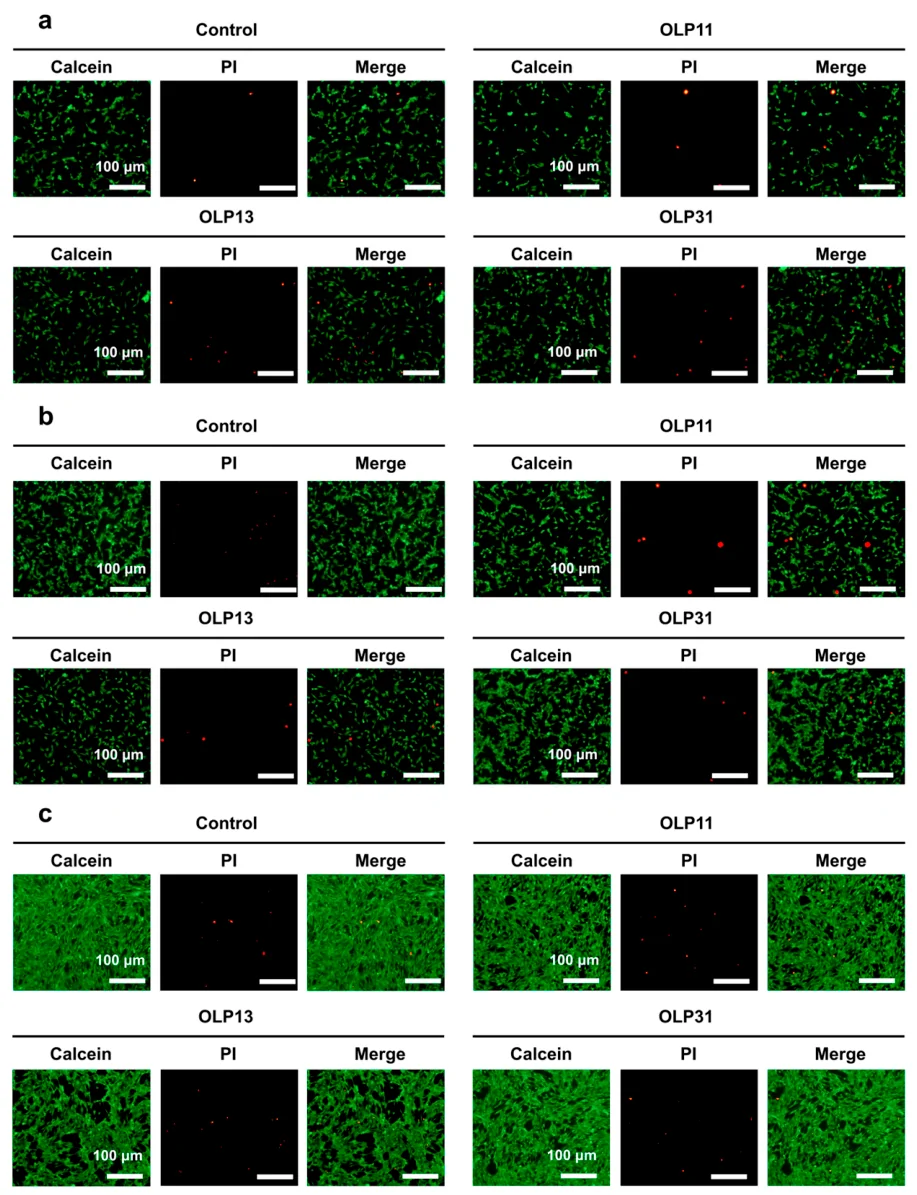
The fluorescence microscope images of NIH 3T3 cells cultured on day 1 (**a**), day 2 (**b**), and day 3 (**c**) using OLP11, OLP13, and OLP31 hydrogel extracts. Calcein-AM/PI staining was used, in which live cells were stained green and dead cells were stained red.

**Figure 8:**
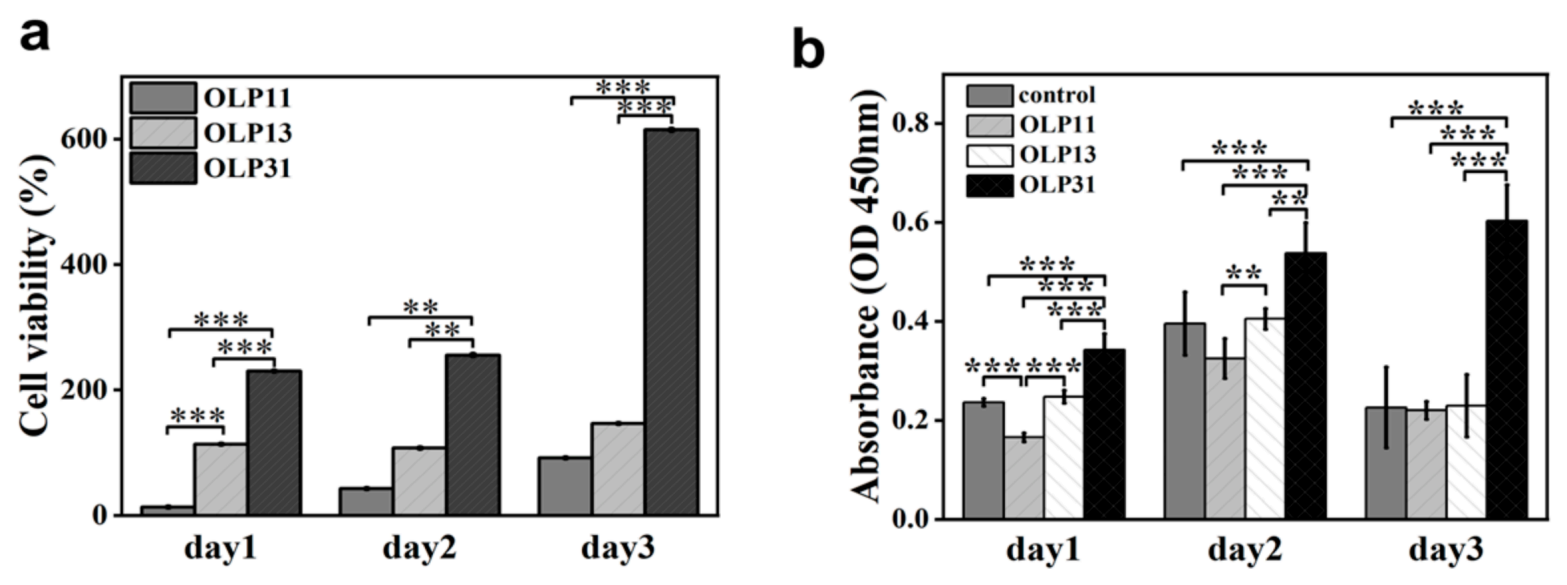
Cytocompatibility of OLP hydrogels evaluated by CCK-8 assay. (**a**) Cell viability of NIH-3T3 cells cultured with OLP11, OLP13, and OLP31 hydrogel extracts for 1, 2, and 3 days. Cells cultured in complete medium without hydrogel treatment served as the control group. (**b**) Corresponding absorbance values (OD 450 nm). ** *p* < 0.01, *** *p* < 0.001.

NIH 3T3 cells were cultured using OLP11, OLP13, and OLP31 hydrogel extracts to evaluate the cytocompatibility of OLP hydrogels. After Calcein-AM/PI staining, the cell status was observed using a fluorescence microscope (Figure 7a–c). A few dead cells (red dots, stained by PI) were observed in all groups, and the live cells (green dots, stained by Calcein-AM) were apparently increased over days. Subsequent cell viability experiments conducted using the CCK-8 assay corroborated the live/dead staining results (Figure 8a,b). Fluorescence images revealed minimal red dead cells across all groups. This indicated negligible cytotoxicity of the hydrogel extracts. The density of living cells steadily increased during the culture period. Notably, OLP31 consistently exhibited the highest cell density. OLP13 showed moderate cell growth, while OLP11 displayed the lowest density.

The quantitative CCK-8 results validated the observations obtained from staining. OLP31 demonstrated the highest cell viability among all groups, followed by OLP13, and OLP11 had the lowest. Moreover, both OLP31 and OLP13 were significantly higher than OLP11. Notably, the cell viability of OLP31 exceeded 600% on day 3, indicating a significant increase in cell viability. Overall, all groups showed a trend of increasing cell viability over time during the culture period.

### 3.8. Cell Pro-Migration Ability of OLP Hydrogels

**Figure 9:**
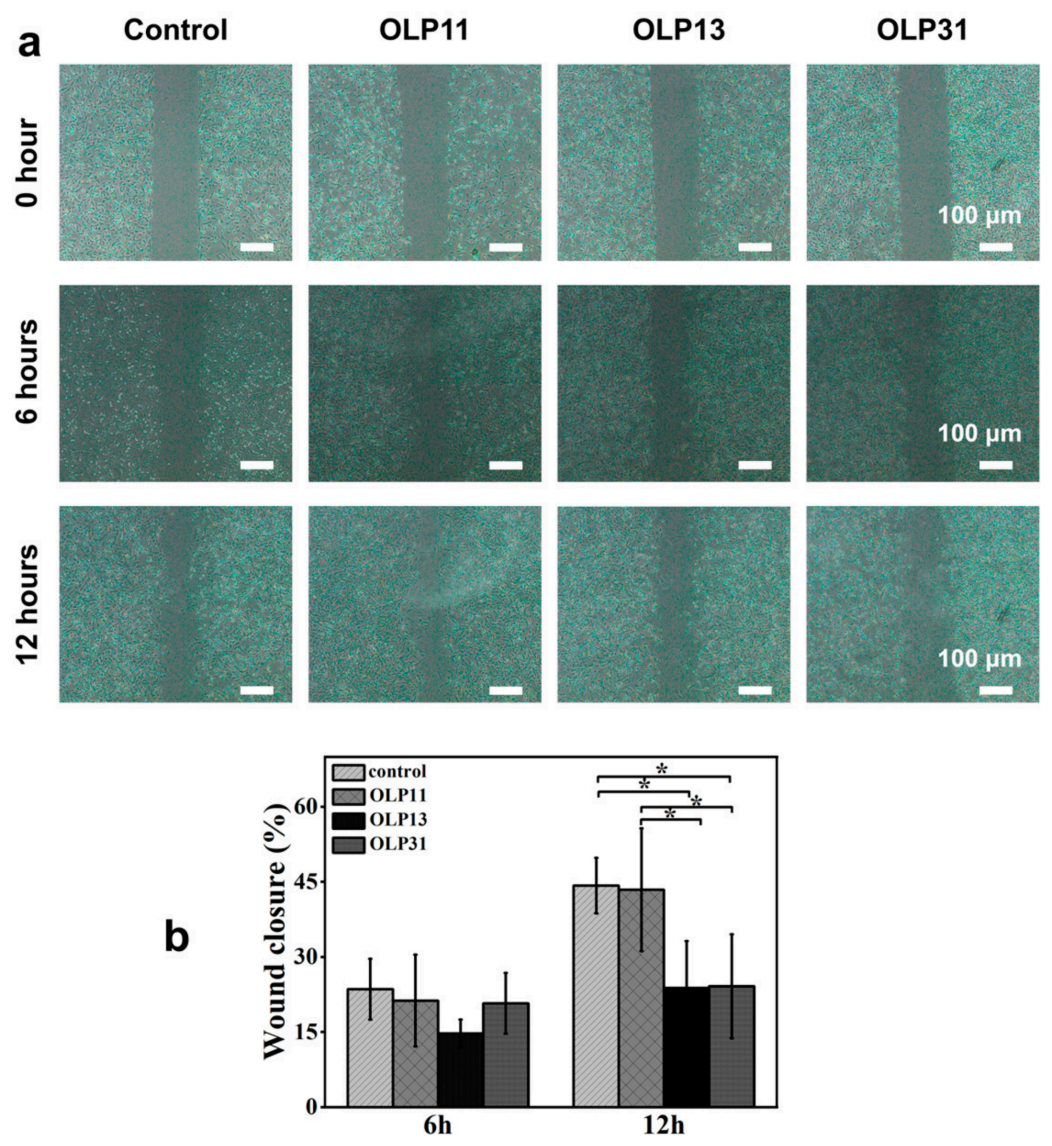
The cell migration promoting ability of OLP hydrogels was evaluated through scratch experiments. (**a**) Optical images of NIH 3T3 cells cultured with OLP11, OLP13, and OLP31 hydrogel extracts, respectively (6 h and 12 h). Cells cultured in complete medium without hydrogel treatment served as the control group. (**b**) The wound closure rates of NIH 3T3 cells. * *p* < 0.05.

The effect of OLP hydrogels on cell migration was evaluated via the scratch assay (Figure 9a). Distinct scratch gaps were visible at 0 h. After 6 h of culture, cells migrated into the cell-free region to drive wound closure. At 12 h, however, the OLP13 and OLP31 hydrogel groups exhibited substantially weaker scratch closure capacity relative to the control and OLP11 groups. Furthermore, migration rates were statistically calculated using ImageJ, and the results are shown in Figure 9b. At 6 h, no significant differences were detected among the groups. At 12 h, both Control and OLP11 groups exhibited high migration rates of approximately 45%. However, the migration rates of OLP13 and OLP31 were significantly lower than those of the Control and OLP11 groups. This indicates that the OLP11 hydrogel prepared with the same mass ratio of ODex and LA-PEI has the best cell migration promoting ability. However, regardless of the incorporated component, the migration promoting ability of the hydrogel was decreased. We speculate that the excess positive charge from PEI in OLP13 may interfere with integrin-mediated cell–matrix interactions, thereby compromising its migration-promoting capacity. For OLP31, the relatively high proportion of oxidized dextran may generate excess free aldehyde groups, which could affect the normal function of cell surface proteins through non-specific interactions, potentially hindering cell migration. In contrast, OLP11 appears to achieve a more favorable compositional balance, avoiding excessive charge interference while maintaining a microenvironment conducive to cell migration, which may account for its best performance in promoting cell migration among the three groups.

## 4. Conclusions

In summary, this study successfully fabricated composite OLP hydrogel adhesives via Schiff-base crosslinking between ODex and LA-PEI. The hydrogels exhibit tunable porous structures, favorable swelling properties, self-healing capability, strong tissue adhesion, and excellent antioxidant, antibacterial, hemocompatibility, and cytocompatibility. While the current study demonstrates promising in vitro results, future work will focus on in vivo wound-healing evaluation using appropriate animal models to further validate the translational potential of the OLP hydrogel system. Notably, the OLP11 formulation shows the best cell migration-promoting effect, while OLP31 significantly enhances cell viability. Therefore, this composite OLP hydrogel bio-adhesive can be tailored for various application scenarios, demonstrating broad potential for biomedical applications.

## Data Availability

Data will be made available on request.
